# Gamma amino butyric acid (GABA) application modulated the morpho-physiological and yield traits of fragrant rice under well-watered and drought conditions

**DOI:** 10.1186/s12870-024-05272-5

**Published:** 2024-06-18

**Authors:** Umair Ashraf, Shakeel Ahmad Anjum, Sidra Naseer, Anees Abbas, Muhammad Abrar, Mohsin Nawaz, Kebo Luo

**Affiliations:** 1 Department of Botany, Division of Science and Technology, University of Education, Lahore, Punjab 54770 Pakistan; 2 Department of Agronomy, University of Agriculture, Faisalabad, Punjab 38040 Pakistan; 3 Department of Botany, Faculty of Sciences, University of Agriculture, Faisalabad, Punjab 38040 Pakistan; 4 State Key Laboratory of Grassland Agroecosystem, School of Life Sciences, Lanzhou University, Lanzhou, China; 5 Institute of Environment and Ecology, School of the Environment and Safety Engineering, Jiangsu University, Zhenjiang, 212013 China; 6 Jieyang Research Institute of Agricultural Sciences, Jieyang, China

**Keywords:** Antioxidants, Drought, Fragrant rice, GABA, Yield

## Abstract

**Background:**

Changing climate is causing erratic rainfall and prolonged drought periods, thus posing serious threats to crop productivity. Owing to severity of drought events, it is imperative to take proactive measures to enhance the resilience of drought sensitive crops like rice. Therefore, the present study was carried out to improve the drought stress tolerance in rice through gamma amino butyric acid (GABA) application.

**Methods:**

The experiment was included four GABA concentrations i.e., 0 mM as control, 1 mM, 1.5 mM, and 2 mM, two water levels i.e., 100% and 50% field capacity (referred as FC100 for well-watered and FC50 for drought conditions, respectively), and two fragrant rice cultivars i.e., Super Basmati and Basmati-515.

**Results:**

The findings unveiled a comprehensive improvement in various parameters with GABA application in fragrant rice under both well-watered (FC100) and water-limited (FC50) conditions, compared to the control. Specifically, GABA induced enhancements were observed in plant height, root length, fresh weight, dry weight, total soluble protein content, and total free amino acid content across both cultivars. Moreover, GABA application significantly improved peroxidase (POD) and catalase (CAT) enzyme activities, alongside elevating anthocyanin levels, while concurrently reducing H_2_O_2_ contents in both FC100 and FC50 treatments. Furthermore, the positive impact of GABA extended to morphological traits, with notable increases in panicle length, total tillers and productive tillers per hill, branch and grain numbers per panicle, and 1000-grain weight for Super Basmati and Basmati 515 cultivars under both water regimes, compared to Ck. Similarly, the grain yield increased by 31.01% and 27.32% under FC100 and 36.85% and 27.71% under FC50 in Super Basmati and Basmati-515, respectively, in response to GABA application, compared to Ck. Additionally, principal component analysis (PCA) revealed significant variances attributed to Dim1 and Dim2, with 86.1% and 4.0% of the variance, respectively, across three bi-plots encompassing rice cultivars, water levels, and GABA treatments. Notably, all tested indices, except for H_2_O_2_ and non-productive tillers per hill, exhibited positive correlations amongst themselves and with rice yield, further emphasizing the beneficial effects of GABA application on fragrant rice under well-watered and drought conditions.

**Conclusions:**

GABA significantly improved fragrant rice performance under both well-watered (FC100) and water-limited (FC50) conditions. Moreover, integrating GABA application into rice cultivation practices could not only improve the crop resilience to drought stress but also potentially benefiting the future food and nutritional security globally. However, however; further research is needed to understand the cellular and molecular mechanisms of the functionality of GABA in fragrant rice, particularly under drought conditions.

**Supplementary Information:**

The online version contains supplementary material available at 10.1186/s12870-024-05272-5.

## Background

Rice (*Oryza sativa* L.) stands as a vital staple for over one-third of the world’s population, serving as a cornerstone of global nutrition due to its richness in carbohydrates, proteins, vitamins, and minerals [1]. Among different rice types, fragrant rice is well-famous among consumers owing to its ‘pop-corn’ like or nutty aroma, taste and premium cooking qualities [2] owing to the presence of aromatic compounds, i.e., 2-acetyl-1-pyrroline (2-AP) [3]. However, despite of excellent grain quality, fragrant rice types are comparatively low yielding than other rice types and also sensitive to water stress or drought conditions [4].

The onset of water deficit conditions can lead to a staggering 50% decrease in crop yield, with the severity escalating to the point of complete crop failure under conditions of severe drought [5]. Physiologically, plants respond to drought stress by producing reactive oxygen species (ROS) as signaling molecules to regulate stress response mechanisms [6], affecting water relations, gas exchange, photosynthesis, and organic compounds metabolism [7]. Excessive ROS production under drought conditions often damages various cell components and reduces photosynthesis, gas exchange attributes, photosystem II (PSII) quantum efficiency, and carboxylation efficiency in rice [8]. Furthermore, ROS can inflict damage on lipids, DNA, proteins and other essential plant organelles [9]. Drought stress causes an imbalance in turgor that results in stomatal closure, further diminishing photosynthesis [10]. Hence, proactive measures are needed to cope with drought stress in rice, employing various approaches such as GABA (Gamma amino butyric acid) application [11]. GABA, known for its drought tolerance-inducing qualities in crops, presents a promising avenue for enhancing rice resilience in the face of water scarcity [12].

GABA, a four-carbon non-protein amino acid, synthesized through the ‘GABA shunt’ pathway, plays a pivotal role in various biological processes [13]. Its multifaceted functions extend to the modulation of gene expression, particularly in response to environmental stresses such as drought [14]. Specifically, GABA treatment upregulates genes involved in reactive oxygen species (ROS) scavenging, such as superoxide dismutase (SOD), catalase (CAT), and ascorbate peroxidase (APX), enhancing the antioxidant defense system in drought-stressed plants [15]. Additionally, genes related to the biosynthesis of osmolytes like proline and glycine betaine are upregulated by GABA application under drought stress [16]. This comprehensive regulatory effect is further evidenced by GABA’s induction of various stress-responsive transcription factors, which orchestrate the expression of numerous drought-responsive genes involved in stress signaling, osmotic adjustment, and other protective mechanisms [17].

Moreover, GABA application promotes the upregulation of stress-protective proteins like dehydrins, safeguarding cellular structures and enzymes from dehydration damage during drought [18]. Furthermore, GABA’s influence extends to hormone signaling pathways, including abscisic acid (ABA), auxins, and ethylene, critical for orchestrating drought stress responses in plants [19]. Interestingly, GABA treatment downregulates the expression of the ABA biosynthesis gene under drought conditions, thereby modulating ABA levels and associated drought responses. Additionally, GABA plays a crucial role in regulating intracellular processes like cell growth and differentiation, enhancing morphological growth, chlorophyll biosynthesis, photosynthetic and gas exchange attributes, and membrane stability in rice [19]. Previous studies have documented the ability of GABA to enhance drought tolerance across various crops [20]. However, limited research has explored the specific effects of GABA-induced modulation of the growth, yield and biochemical traits of fragrant rice under normal and water stress conditions. Therefore, this study aims to fill this gap by investigating the impact of GABA application on the on the performance of fragrant rice in both well-watered and drought conditions, thus offering insights into its potential as a strategy for enhancing fragrant rice resilience against drought stress. Integrating GABA application at different growth stages depending upon the onset of drought period and/or water shortage in rice could mitigate the adverse impacts of drought on rice growth and productivity, making it a critical tool for future agronomic strategies and climate adaptation efforts.

## Methods

### Experimental details

A pot experiment was conducted at the University of Agriculture, Faisalabad (UAF), Pakistan (31.45° N, 73.14° E; altitude, 186 m) in 2018, under natural conditions in rain-protected shelter house. Seeds of two fragrant rice cultivars (Super Basmati and Basmati-515) were obtained from the Rice Research Institute, Kala Shah Kaku, Punjab, Pakistan. These cultivars are widely known and cultivated as ‘Basmati rice’ across Punjab Province and are both locally consumed and exported internationally [21]. Soil for the experiment was collected from a field under a rice–wheat cropping system at the Agronomic Research Area, UAF. Each plastic pot (33 cm height × 28 cm diameter) was filled with 12 kg of air-dried soil. The physicochemical properties of the experimental soil are described in Table 1.

**Table 1:** Physicochemical properties of the experimental soil

Parameter	Value/Status
Soil pH	7.8
Soil EC (dSm^− 1^)	1.12
Organic matter (%)	0.56
Soil bulk density (mg m^− 3^)	1.41
N content (%)	0.032
Phosphorous (mg kg^− 1^ soil)	24
Available potassium (mg kg^− 1^ soil)	23
Total Boron (mg kg^− 1^ soil)	0.61
Total Zinc (mg kg^− 1^ soil)	1.32
Soil type	Sandy-Clay-loam

Seeds of both rice cultivars were sown on June 21, 2018, for nursery development. After one month, two seedlings per hill and three hills per pot were transplanted. The experimental treatments included four GABA concentrations: 0 mM (control; Ck), 1 mM (G1), 1.5 mM (G2) and 2 mM (G3), along with two water levels: 100% field capacity (FC100, well-watered) and 50% field capacity (FC50, drought). After 30 days of transplanting, drought was initiated and continued for 22 days, spanning from post-tillering to the booting stage. This drought treatment, imposed by withholding water, required the pots to be re-watered after being weighed on daily basis during this phase to maintain their respective field capacity [22]. GABA application [23] began one week after withholding water, with foliar application was employed by handheld atomizer till complete wetting of all leaves and repeated for two consecutive days to make sure effectiveness of the GABA. Control pots were sprayed with the distilled water instead of GABA. Additionally, 25 g of N, 1.75 g of P and 1.35 g of K were applied to each pot whereas all the other agronomic practices were kept uniform, except for the experimental treatments. Plants were harvested manually on November 10, 2018.

### Observations and data collection

#### Morphological traits

At physiological maturity, measurements of plant height, root length, and fresh and dry root biomass were recorded. The plants were carefully removed from the pots with their roots intact and washed thoroughly. Plant height and root length were measured using a meter rod, while roots were separated from plants and weighed to record their fresh weight. Subsequently, the fresh roots were oven-dried at 60 °C until a constant weight was reached to determine the dry weight.

### Biochemical attributes

Fresh leaves were cut two weeks after GABA application (before ending the drought treatment), during the booting stage. These leaves were thoroughly washed and then stored at ‒80 °C for biochemical assays. These leaves (0.5 g) were homogenized in 5 ml of sodium phosphate buffer (pH 7.8) and centrifuged at 10,000 rpm for 5 min. The resulting supernatant was collected for biochemical analysis. The total soluble protein concentration was determined according to Bradford [24]. The 2 ml of Bradford solution was added to 0.1 ml of crude extract and vortexed. The absorption of the reaction mixture was recorded at 595 nm. The total soluble protein contents were expressed as mmol g^− 1^ fresh weight (FW). Total free amino acids were estimated according to Hamilton, et al. [25]. Briefly, 1.0 ml of 2% ninhydrin and 1.0 ml of 10% pyridine solution were added to 1.0 ml of crude extract, heated at 90 °C for 30 min in a water bath, and cooled. The absorbance of the mixture was read at 570 nm and is expressed as mg g^− 1^ fresh weight (FW). The H_2_O_2_ contents were determined according to Velikova, et al. [26]. Briefly, fresh leaves (0.5 g) were homogenized in 5 ml of 5% trichloroacetic acid (TCA) and centrifuged at 10,000 rpm for 5 min. One ml of 1 M KI and 100 µl of phosphate buffer (pH 7.8) were added to 1 ml of supernatant, and the absorbance was recorded at 390 nm and is expressed as mmol g^− 1^ FW. The peroxidase (POD) activity was measured according to Chance and Maehly [27]. One ml of potassium buffer (pH 7.0), 100 µl of 0.2% guaiacol, 100 µl of 0.3% H_2_O_2_, and 100 µl of supernatant were mixed, and the absorbance was read at 470 nm. One unit of POD activity (U) was the amount of enzyme that caused oxidation of the substrate in the presence of H_2_O_2_. The catalase (CAT) activity was determined according to Aebi [28]. Briefly, 2.0 ml of sodium phosphate buffer (pH 7.8), 0.1 ml of H_2_O_2_ (0.1 M), and 0.1 ml of supernatant were mixed, and the absorbance was measured at 240 nm. One unit of enzyme activity (U) was defined as the decomposition of 1 M H_2_O_2_ at A_240_ within 1 min in 1 g of fresh leaf sample. The anthocyanin content was assessed according to Fuleki and Francis [29], and the absorbance was read at 535 nm. The anthocyanin contents are expressed as mmol g^− 1^ fresh weight (FW). The absorbance values for all biochemical parameters were obtained by using a double-beam UV/Vis spectrophotometer (Shimadzu, Japan).

### Yield and yield attributes

Total tillers and productive (panicle-bearing), and non-productive tillers were counted at the active tillering and panicle heading stages, respectively. At physiological maturity, all remaining pots were harvested manually, and measurements including panicle length, branch and grain numbers per panicle, 1000-grain weight, grain yield, and straw yield were recorded.

### **Experimental design and statistical analysis**

The pots were arranged in a completely randomized design (CRD) with five pots per treatment and three replications of each experimental unit, resulting in a total of 15 pots per treatment overall. The data were analyzed statistically using three-way analysis of variance (ANOVA) with SPSS 22.0 software (SPSS, Chicago, IL, USA). Treatment means were compared using Tukey’s honest significant difference (HSD) test (*p < 0.05*). Figures were generated using OriginLab 2021 software (Origin Lab Corporation, USA).

## Results

### Morphological attributes

**Fig. 1:**
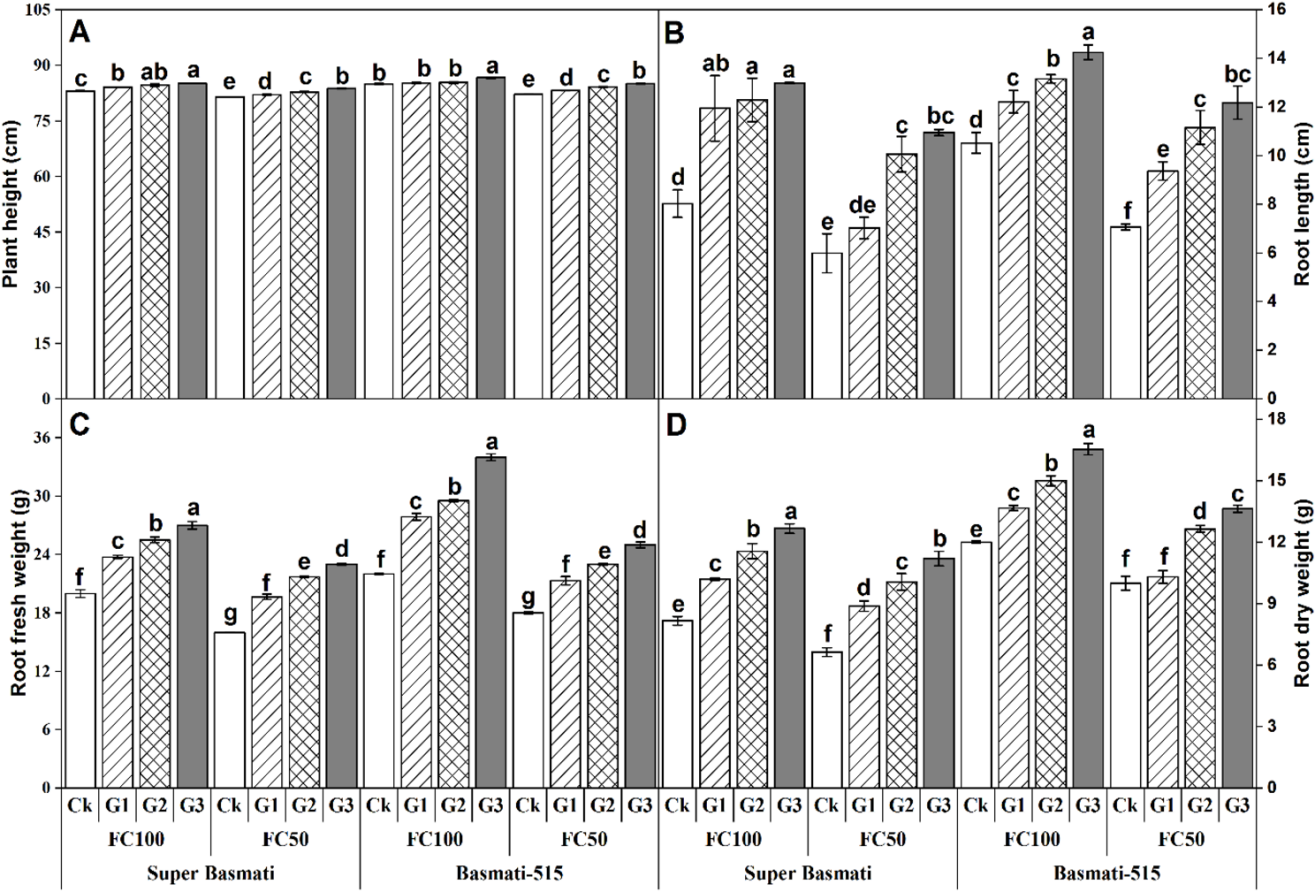
Effect of foliar application of GABA on plant height **(A)**, root length **(B)**, root fresh weight **(C)** and root dry weight **(D)** of two fragrant rice cultivars (Super Basmati and Basmati-515) under well-watered (FC100) and drought (FC50) conditions. Vertical bars with different lowercase letters for each cultivar are significantly different according to Tukey’s HSD test (*p < 0.05*). The error bars above the means are the standard errors (Means ± SE) of three replicates (*n* = 3). Ck (control), G1, G2 and G3 indicate 0, 1, 1.5 and 2 mM GABA application, respectively (the same in the blew)

GABA application significantly (*p < 0.5*) affected the plant height, root length, and root fresh and dry weight of both rice cultivars grown under the well-watered (FC100) and drought (FC50) conditions (Table 2). For Super Basmati, compared to the control (Ck), GABA application led to an increase in plant height, root length, root fresh weight, and root dry weight by 2.43%, 61.87%, 35% and 55.20%, respectively, in the FC100 treatment whereas the plant height, root length, root fresh weight, and root dry weight was increased by 2.75%, 82.75%, 43.75% and 68.69%, respectively under the FC50 treatment. Similarly, for Basmati 515, GABA application resulted in an increase in plant height, root length, root fresh weight and root dry weight by 2.01%, 35.40%, 54.54%, and 37.78%, respectively, in the FC100, and by 3.47%, 72.32%, 38.88% and 36.35%, respectively, in the FC50 treatment compared to control (Fig. 1).

**Table 2:** 3-way ANOVA (Analysis of variance) for morpho-biochemical and yield traits of two fragrant rice cultivars treated with GABA application under well-watered and drought conditions

Traits	GABA (G)	Water levels (W)	Varieties (V)	G × W	G × V	W × V	G × W × V
Plant height	< 0.0001 (0.96)	< 0.0001 (0.97)	< 0.0001 (0.93)	0.0002 (0.46)	0.1133 (0.17)	0.1119 (0.08)	< 0.0001 (0.50)
Root length	< 0.0001 (0.89)	< 0.0001 (0.83)	< 0.0001 (0.54)	0.0172 (0.27)	0.6152 (0.05)	0.6383 (0.01)	0.0605 (0.20)
Root Fresh weight	< 0.0001 (0.99)	< 0.0001 (0.98)	< 0.0001 (0.95)	< 0.0001 (0.54)	< 0.0001 (0.69)	< 0.0001 (0.69)	< 0.0001 (0.52)
Root dry weight	< 0.0001 (0.95)	< 0.0001 (0.89)	< 0.0001 (0.95)	0.8261 (0.03)	0.0020 (0.36)	< 0.0001 (0.42)	0.5701 (0.06)
Total soluble proteins	< 0.0001 (0.97)	< 0.0001 (0.96)	< 0.0001 (0.89)	< 0.0001 (0.65)	< 0.0001 (0.54)	0.0517 (0.11)	0.1525 (0.15)
Total free amino acids	< 0.0001 (0.99)	< 0.0001 (1.00)	< 0.0001 (0.96)	< 0.0001 (0.93)	< 0.0001 (0.57)	0.9854 (0.00)	< 0.0001 (0.54)
Hydrogen peroxide	< 0.0001 (0.97)	< 0.0001 (0.96)	0.0105 (0.19)	< 0.0011 (0.39)	0.1627 (0.15)	< 0.0001 (0.82)	0.0075 (0.31)
Peroxidase	< 0.0001(0.93)	< 0.0001 (0.94)	< 0.0001 (0.75)	0.0023 (0.36)	0.0164 (0.27)	< 0.0001 (0.47)	0.9041 (0.02)
Catalase	< 0.0001 (1.00)	< 0.0001 (1.00)	< 0.0001 (0.99)	< 0.0001 (0.97)	< 0.0001 (0.88)	< 0.0001 (0.92)	< 0.0001 (0.88)
Anthocyanin	< 0.0001 (0.88)	< 0.0001 (0.97)	< 0.0001 (0.79)	0.0688 (0.20)	< 0.0001 (0.57)	0.6554 (0.01)	0.1814 (0.14)
Total tillers plant^**-1**^	< 0.0001 (0.85)	< 0.0001 (0.88)	0.0014 (0.28)	0.0228 (0.25)	0.42099 (0.08)	< 0.0001 (0.46)	0.3957 (0.09)
Productive tillers plant^**-1**^	< 0.0001 (0.87)	< 0.0001 (0.95)	< 0.0001 (0.77)	0.0490 (0.21)	0.0672 (0.20)	0.2744 (0.04)	0.4495 (0.08)
Nonproductive tillers plant^**-1**^	0.0214 (0.26)	< 0.0001 (0.91)	< 0.0001 (0.85)	0.7908 (0.03)	< 0.0001 (0.65)	< 0.0001 (0.67)	0.7668 (0.03)
Panicle length	< 0.0001 (0.66)	< 0.0001 (0.82)	< 0.0001 (0.48)	0.4375 (0.08)	0.9175 (0.02)	0.0298 (0.14)	0.2016 (0.13)
Branches panicle^**-1**^	< 0.0001 (0.99)	< 0.0001 (0.99)	< 0.0001 (0.98)	< 0.0001 (0.66)	< 0.0001 (0.62)	0.0795 (0.09)	< 0.0001 (0.89)
Grains panicle^**-1**^	< 0.0001 (0.85)	< 0.0001 (0.94)	< 0.0001 (0.77)	0.0172 (0.27)	0.1421 (0.15)	< 0.0001 (0.49)	0.1939 (0.14)
1000-grain weight	< 0.0001 (0.86)	< 0.0001 (0.95)	< 0.0001 (0.90)	0.2602 (0.12)	0.1080 (0.17)	0.1148 (0.08)	0.9377 (0.01)
Grain yield	< 0.0001 (0.65)	< 0.0001 (0.52)	0.0784 (0.09)	0.9342 (0.01)	0.7886 (0.03)	0.0401 (0.13)	0.9977 (0.00)
Straw yield	< 0.0001 (0.92)	< 0.0001 (0.96)	< 0.0001 (0.73)	< 0.0001 (0.50)	0.0114 (0.29)	0.0002 (0.35)	0.0247 (0.25)

The digits represent probability values derived from the ANOVA table and the digits in parenthesis indicate partial eta-square (η^2^) values

### Biochemical attributes

**Fig. 2:**
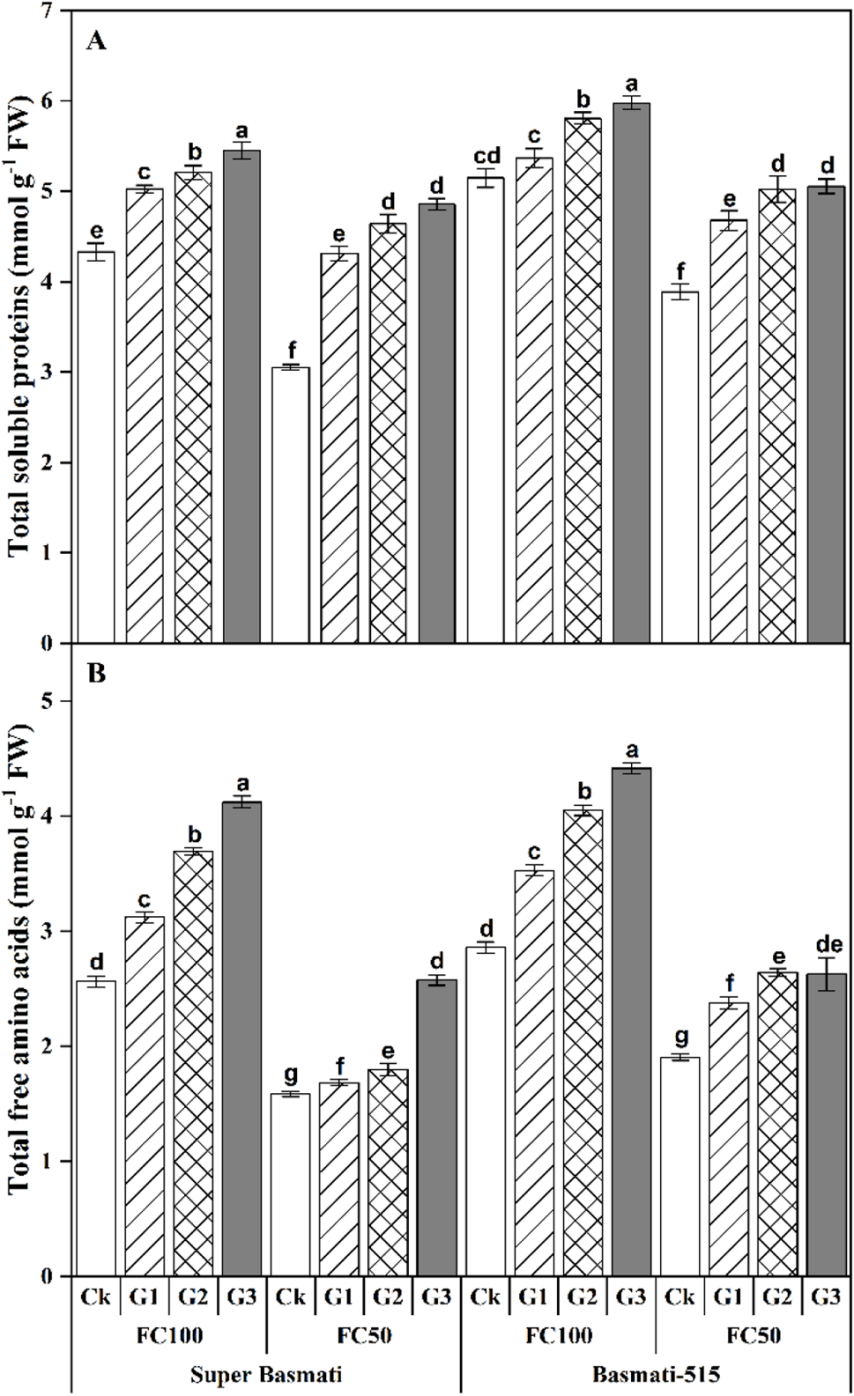
Effect of foliar application of GABA on total soluble proteins **(A)** and total free amino acids **(B)** in the leaves of two fragrant rice cultivars (Super Basmati and Basmati-515) under well-watered (FC100) and drought (FC50) conditions

GABA significantly (*p < 0.5*) affected the total soluble proteins and total free amino acids in both rice cultivars subjected to drought stress (Table 2; Fig. 2A and B). Similarly, compared to control (Ck), the total soluble protein content in Super Basmati increased up to 25.96% under FC100 and 58.98% under FC50 following GABA application. In Basmati-515, the total soluble protein content increased up to 16.09% under FC100 and 30.01% under FC50 in response to GABA application (Fig. 2A). Similarly, the total free amino acid content showed a parallel trend to the total soluble protein content. In Super Basmati, the total free amino acids were increased up to 60.79% under FC100 and 62.40% under FC50 compared to control (Ck). In Basmati-515, the total free amino acid content was enhanced by 24.63% under FC100 and by 37.65% under FC50 after GABA application (Fig. 2B).

**Fig. 3:**
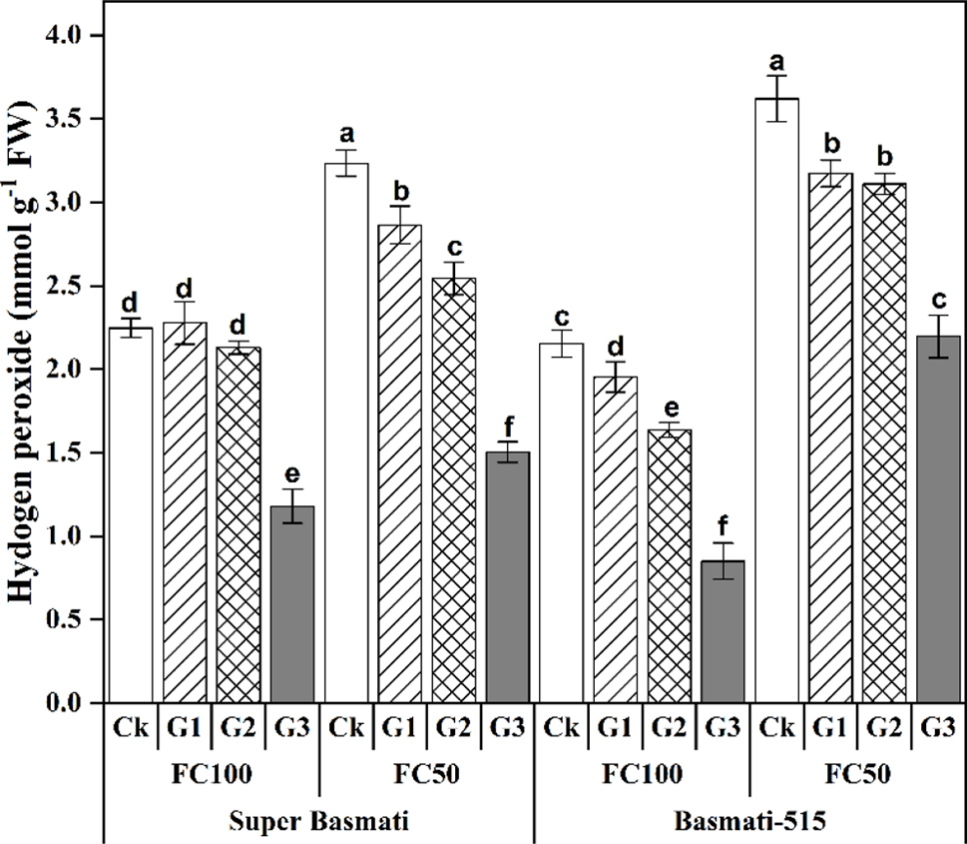
Effect of foliar application of GABA on H_2_O_2_ concentration in the leaves of two fragrant rice cultivars (Super Basmati and Basmati-515) under well-watered (FC100) and drought (FC50) conditions

Furthermore, the hydrogen peroxide (H_2_O_2_) contents were significantly (*p < 0.5*) higher in both rice cultivars under FC50. However, a significant reduction in H_2_O_2_ content was observed with GABA application under both FC100 and FC50 treatments. Specifically, GABA treatment reduced the H_2_O_2_ content by 47.48% and 53.50% in Super Basmati and by 60.52% and 39.31% in Basmati-515 under FC100 and FC50, respectively (Fig. 3).

**Fig. 4:**
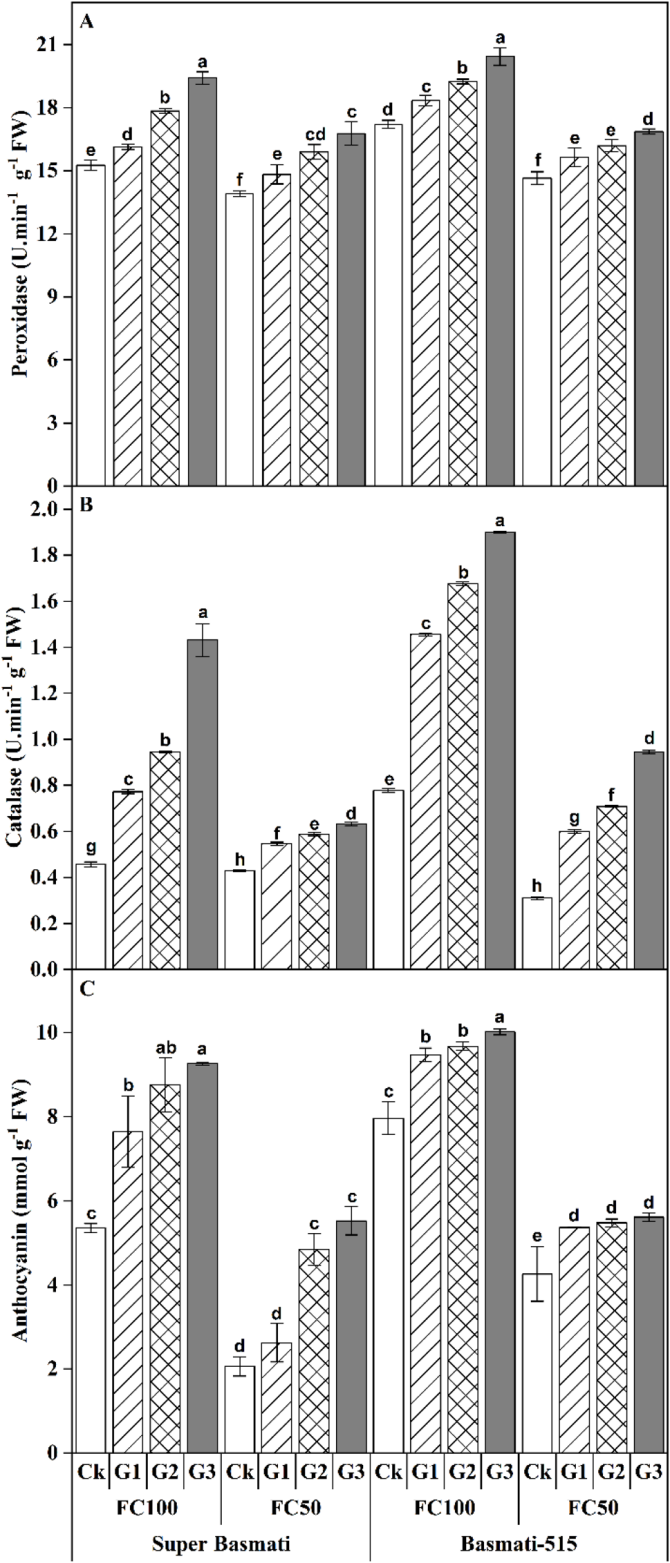
Effect of foliar application of GABA on peroxidase **(A)**, catalase **(B)** and anthocyanin content **(C)** in the leaves of two fragrant rice cultivars (Super Basmati and Basmati-515) under well-watered (FC100) and drought (FC50) conditions

Additionally, GABA application significantly (*p < 0.5*) affected peroxidase (POD) and catalase (CAT) activities as well as anthocyanin contents in both rice cultivars under the FC100 and FC50 treatments (Table 2). The POD activity increased with increasing GABA concentration under both water levels for both rice cultivars, although POD activity remained lower in FC50 treatment compared to FC100 treatment. In Super Basmati, the POD activity increased up to 27.21% under FC100 and 20.60% under FC50 following GABA application. In Basmati-515, POD activity increased up to 18.76% under FC100 and 15.10% under FC50 in response to GABA application. Similarly, a gradual increase in GABA application resulted in increased CAT activity in both rice cultivars under both water treatments, with CAT activity being lower under FC50 than under FC100 (Fig. 4A). Specifically, the CAT activity increased by 213.12% and 144.38% under FC100, and by 47.51% and 204.47% under FC50 with GABA application compared to control (Ck) treatment in both Super Basmati and Basmati-515, respectively (Fig. 4B). Furthermore, GABA application also led to increased anthocyanin content, with Super Basmati showing increase of 72.95% under FC100 and 168.02% under FC50, and Basmati-515 showing increase of 25.74% under FC100 and 31.66% under FC50 (Fig. 4C).

### Yield and yield attributes

**Fig. 5:**
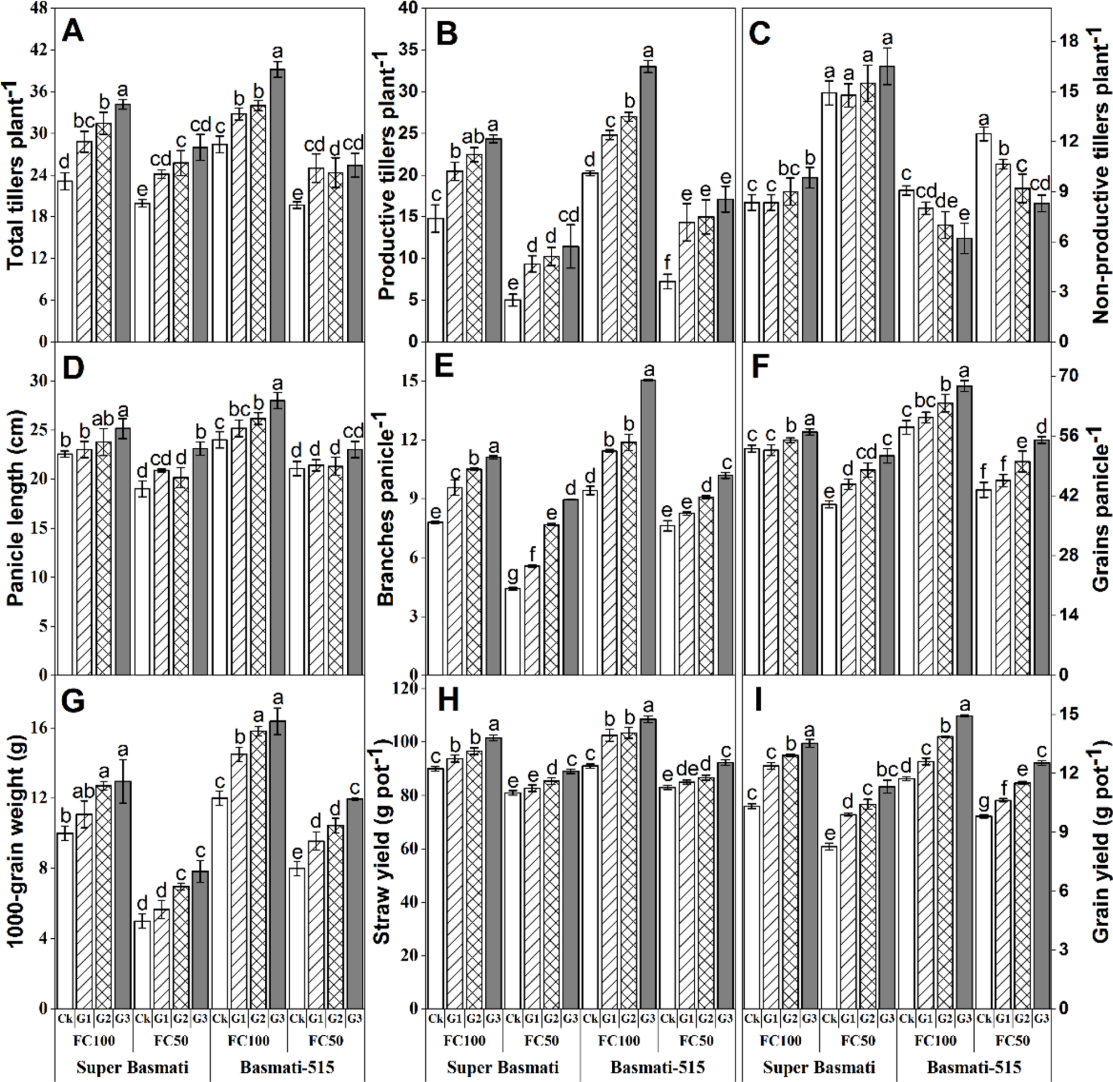
Effect of foliar application of GABA on total tillers plant^− 1^**(A)**, productive tillers plant^− 1^**(B)**, nonproductive tillers plant^− 1^**(C)**, panicle length **(D)**, branch panicle^− 1^**(E)**, grain panicle^− 1^**(F)**, 1000-grain weight **(G)**, straw yield pot^− 1^**(H)** and grain yield pot^− 1^**(I)** in two fragrant rice cultivars (Super Basmati and Basmati-515) under well-watered (FC100) and drought (FC50) conditions

GABA application significantly (*p < 0.5*) affected the yield indices of both fragrant rice cultivars (Table 2; Fig. 5). In Super Basmati, the GABA application improved various yield parameters under both water levels. The panicle length, total tillers per hill, number of productive tillers per hill, number of branches per panicle, number of grains per panicle, and 1000-grain weight were increased by 11.57%, 47.83%, 64.66%, 42.40%, 7.39%, and 29.6% under FC100 and 21.63%, 40.38%, 128.95%, 102.71%, 28.33%, and 56.60%. under FC50, respectively. Comparatively, Super Basmati produced more non-productive tillers than Basmati-515. Nevertheless, GABA application also substantially improved panicle length, total tillers per hill, productive tillers per hill, number of branches per panicle, number of grains per panicle, and 1000-grain weight increased by 16.66%, 37.97%, 63.18%, 59.76%, 16.67%, and 36.5% under FC100 and 9.14%, 29.04%, 136.93%, 33.72%, 26.86%, and 49.37% under FC50, respectively, compared to control (Ck).

In addition, exogenous GABA application led to a significant increase in grain yield at both water levels in both rice cultivars. The grain yield was increased by 31.01 and 36.85% in Basmati-515 and 27.32% and 27.71% in Basmati-515 under FC100 and FC50, respectively, compared to control (Ck) (Fig. 5).

### Principal component analysis

**Fig. 6:**
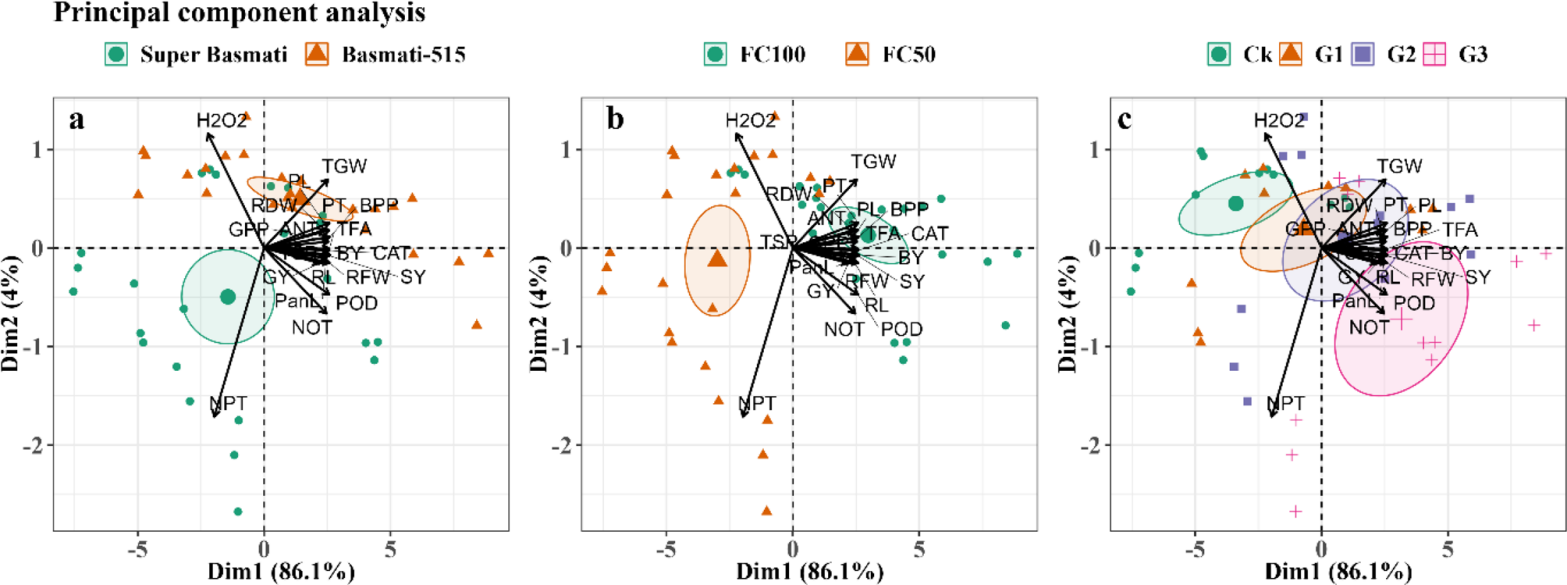
Principal component analysis of the different morpho-biochemical and yield traits of two rice cultivars **(a)** under drought conditions **(b)** treated with foliar application of GABA **(c)**. FC100 and FC50 indicate well-watered and drought conditions, respectively. Ck (control), G1, G2 and G3 indicate 0, 1, 1.5 and 2 mM GABA application, respectively. PH; Plant height, RL; Root length, RFW; Root fresh weight, RDW; Root dry weight, TSP; Total soluble proteins, TFA; Total free amino acids, H_2_O_2_; Hydrogen peroxide, POD; Peroxidase, CAT; Catalase, ANT; Anthocyanin, NOT; Number of total tillers plant^− 1^, PT; Number of productive tillers plant^− 1^, NPT; number of non-productive tillers plant^− 1^, PanL; Panicle length plant^− 1^, BPP; Number of branches panicle^− 1^, GPP; Grains panicle^− 1^, TGW;1000-grain weight, SY; Straw yield, GY; grain yield

The Principal Component Analysis (PCA) revealed that Dim1 accounted for 86.1% of variance, while Dim2 accounted for 4.0% across three bi-plots representing rice cultivars, water level, and GABA treatment. The analysis indicated that the morphological and yield attributes of the rice cultivars were generally positively correlated. In contrast, H_2_O_2_ levels and non-productive tillers were negatively related to catalase (CAT), peroxidase (POD), total free amino acids, and anthocyanin contents. Among the cultivars, Basmati-515 displayed a clustering of plant and yield traits, antioxidant enzymes, and total free amino acids, suggesting greater drought tolerance for this cultivar whereas Super Basmati did not show a clear clustering pattern. In terms of water levels, the well-watered treatment was associated with positive correlations in morphological and yield traits as well as antioxidant enzyme activities. Regarding GABA treatments, the G2 treatment (presumably at a specific concentration) was significantly correlated with a major clustering of plant traits and antioxidants activity, indicating a strong effect on these parameters. In contrast, the G1 and G3 treatments did not show similar clustering patterns which suggests that the G2 treatment had a more pronounced positive impact on the morphological and biochemical attributes of both rice cultivars under the given conditions (Fig. 6).

## Discussion

The effects of GABA application on the morpho-physiological and yield traits of two rice cultivars subjected to drought were assessed. Generally, drought exacerbates growth- and yield-related physio-biochemical mechanisms in plants [16]. However, GABA application can significantly improve the growth and yield of plants under drought stress conditions [30]. In the present study, GABA application substantially enhanced the morphological attributes of both rice cultivars subjected to drought stress, including plant height, root length, and fresh and dry root biomass (Table 2; Fig. 1). These findings align with previous reports by Luo, et al. [31], which showed that exogenous GABA application improved shoot and root growth, photosynthesis, gas exchange, chlorophyll biosynthesis, enzymatic and non-antioxidant defense mechanisms, and membrane stability in tomato plants. These enhancements suggest that GABA aids in maintaining overall plant structure and functioning under stress conditions.

Moreover, GABA application significantly improved the total soluble protein and total free amino acid content while reduced the H_2_O_2_ content in both rice cultivars under both well-watered and drought conditions (Figs. 2 and 3). Previous studies reported that foliar application of GABA improved drought resistance in creeping bent grass (*Agrostis stolonifera*) through the accumulation of amino acids i.e., aspartic acid, glutamic acid, alanine, threonine, valine and serine, organic acids i.e., malic acid, oxalic acid, aconitic acid, threonic acid, and succinic acid, sugars i.e., glucose, sucrose, fructose, maltose, and galactose, sugar alcohols i.e., mannitol and myo-inositol and proline [32]. Additionally, GABA application regulates the biosynthesis and accumulation of secondary metabolites including proline, glutamate, polyamines including putrescine, spermine and spermidine, amino acids in plants [16]. These biochemical changes likely to contribute in enhanced stress tolerance by improving cellular functioning and recovering oxidative damage. In line with our findings, GABA application at 1.0 and 2.0 mg l^− 1^ increased soluble sugar levels by 14.5% and 19.9%, respectively, under water deficit conditions [33]. Similarly, Xie, et al. [34] reported that GABA application enhanced the assimilation of mineral nutrients and modulated aroma biosynthesis in fragrant rice and further reported that GABA application at 250 mg l^− 1^ enhanced the Mn, Fe, and Zn concentration the in grains whereas associations between GABA and N uptake was also found significant and positive of fragrant rice. This suggests that exogenous GABA treatment potentially enhanced nitrogen uptake, leading to increased total soluble proteins and amino acids in both rice cultivars.

GABA regulates levels of sugars and sugar phosphates by stimulating the enzymes involved in tricarboxylic acid (TCA) cycle, thus affects the key intermediates like pyruvate and α-ketoglutarate under stress conditions [13]. High endogenous GABA levels enhanced the cytosolic Ca^2+^ and K^+^ levels by stimulating the osmosensor in the plasma membrane and mediated Na^+^ efflux by modulating the *SOS* genes and *NHX* antiporters [35]. Kinnersley and Lin [36] also reported high Mn and Zn levels in GABA treated *Lemna minor* plants.

Proline transforms into 2-AP by the action of proline dehydrogenase enzyme. GABA induced modulation in endogenous proline contents contributes to the high 2-AP levels whereas higher proline accumulation (as a secondary metabolite) under stress conditions possibly the reason of strong aroma in fragrant rice under stress conditions [34].

In addition, glutamate catalyzed into GABA via decarboxylation reaction indicating a direct relation between them. Moreover, nitrogen in ammonium ions can be converted into glutamate and other amino acids via glutamine synthetase/glutamate synthetase (GS/GOGAT) cycle in cytoplasm whilst in plastids, the glutamate and 2-ketoglutarate also synthesized in plastids as well [37]. In addition, accumulation of several amino acids i.e., Tyr, Asn, Trp, Arg, Lys, and GABA were increased in the seeds of *Arabidopsis* during desiccation phase [38].

Conversely, Aouz, et al. [39] observed that lower soluble protein and free amino acid levels under drought stress could be due to a decrease in photosynthetic activity. Furthermore, the increase in protein and amino acid levels resulting from GABA application may be attributed to the GABA-induced mobilization of starch and amino acids, as reported by Cheng, et al. [40]. These findings are supported by earlier studies on carpet grass and *Portulaca oleracea*, which found that concentrations of soluble proteins and other organic molecules were lower under drought stress compared to normal conditions [7]. On the other hand, the expression of different proteins i.e., GAD2, GABA-T1 and GABA-T2 were increased in kernels after genetic manipulation of GABA shunt in rice [41].

Drought stress often results in increased production of reactive oxygen species (ROS), causing oxidative stress in plant cells [42]. In this study, GABA application significantly improved peroxidase (POD) and catalase (CAT) activities, increased anthocyanin contents, and reduced hydrogen peroxide (H_2_O_2_) levels in both rice cultivars under both well-watered and drought conditions (Figs. 3 and 4). Drought-induced osmotic stress increases the expression of the *OsCIPK02*, *OsCIPK07* and *OsCIPK0* genes, which inhibits rice growth and development [43].

GABA treatment counteracts ROS accumulation, as demonstrated in maize, where it protected cellular membranes from damage, highlighting the role of GABA role in maintaining cell integrity [44]. Similarly, GABA application in rice under heat stress improved osmolytes accumulation and upregulated antioxidant activities [45]. Rezaei-Chiyaneh, et al. [33] reported a significant increase in CAT activity due to GABA application in *Nigella sativa*.

Moreover, anthocyanin play a crucial role in helping plants to endure drought damage during active growth period, with increased anthocyanin production being an adaptive response to drought stress [16]. Additionally, GABA application has been shown to regulate antioxidant defense and photosynthesis in *Capsicum annuum* plants under low light stress [46]. Our findings also revealed positive correlations between total soluble proteins, total free amino acids, POD, CAT, and anthocyanin content with GABA application (Fig. 6). These results underscore the multifaceted benefits of GABA in enhancing plant resilience to drought stress by improving cellular integrity, boosting antioxidant defenses, and promoting key physiological processes.

GABA plays a critical role in multiple metabolic processes within different organelles. For instance, in mitochondria, GABA is converted into succinate via the GABA shunt, which then enters the tricarboxylic acid (TCA) cycle or another metabolic pathway to produce γ-hydroxybutyric acid [47]. Additionally, drought stress triggers Ca^2+^ signal transduction in plants, which activates glutamic acid decarboxylase activity to produce GABA. this compound helps in drought stress tolerance by modulating osmolyte accumulation, maintaining leaf turgor, and reducing oxidative stress through the regulation of the antioxidant defense system [16]. Under drought stress, increased ROS production activates glutamate dehydrogenase (GDH), promoting the conversion of α-ketoglutarate to glutamate, a precursor of GABA [48]. While, the roles of GABA in improving antioxidant activities is well documented, it remains unclear whether this role is direct or indirect. Bouche and Fromm [49] suggested that GABA degradation could limit ROS production under stress conditions. Evidence for this hypothesis comes from the studies on mutant yeast, where the absence of GABA-shunt genes resulted in sensitivity to H_2_O_2_, highlighting potential role of GABA in activating the antioxidant defense system to scavenge ROS. Furthermore, proline-GABA transporters aid plants in accumulating various osmolytes and compatible solutes, providing protection against water stress conditions [49]. Drought stress promotes the degradation of polyamines into GABA due to the activation of GABA-related enzymes [50]. Additionally, high concentrations of cytosolic Ca^2+^ ions activates calmodulin-dependent glutamate decarboxylase, leading to increased GABA synthesis under multiple stress conditions [51].

Drought stress severely affects rice yield and its contributing traits, however, GABA application improved yield traits in both rice cultivars under both well-watered and drought conditions (Fig. 5). Rice yield is directly associated with the number of productive tillers, branches per panicle, panicle length, and grains per panicle, making these key traits critical for improving rice productivity [12]. In this study, GABA-treated plants exhibited improved productive tillers, panicle length, number of branches per panicle, and number of grains per panicle, likely resulting in higher grain yield. Generally, drought-tolerant rice cultivars produce more roots than drought-sensitive ones, which contributes to better grain weight and source‒sink relationships under drought conditions [16]. Additionally, GABA has been shown to significantly improve wheat yield by enhancing yield-contributing attributes under drought stress conditions [17].

**Fig. 7:**
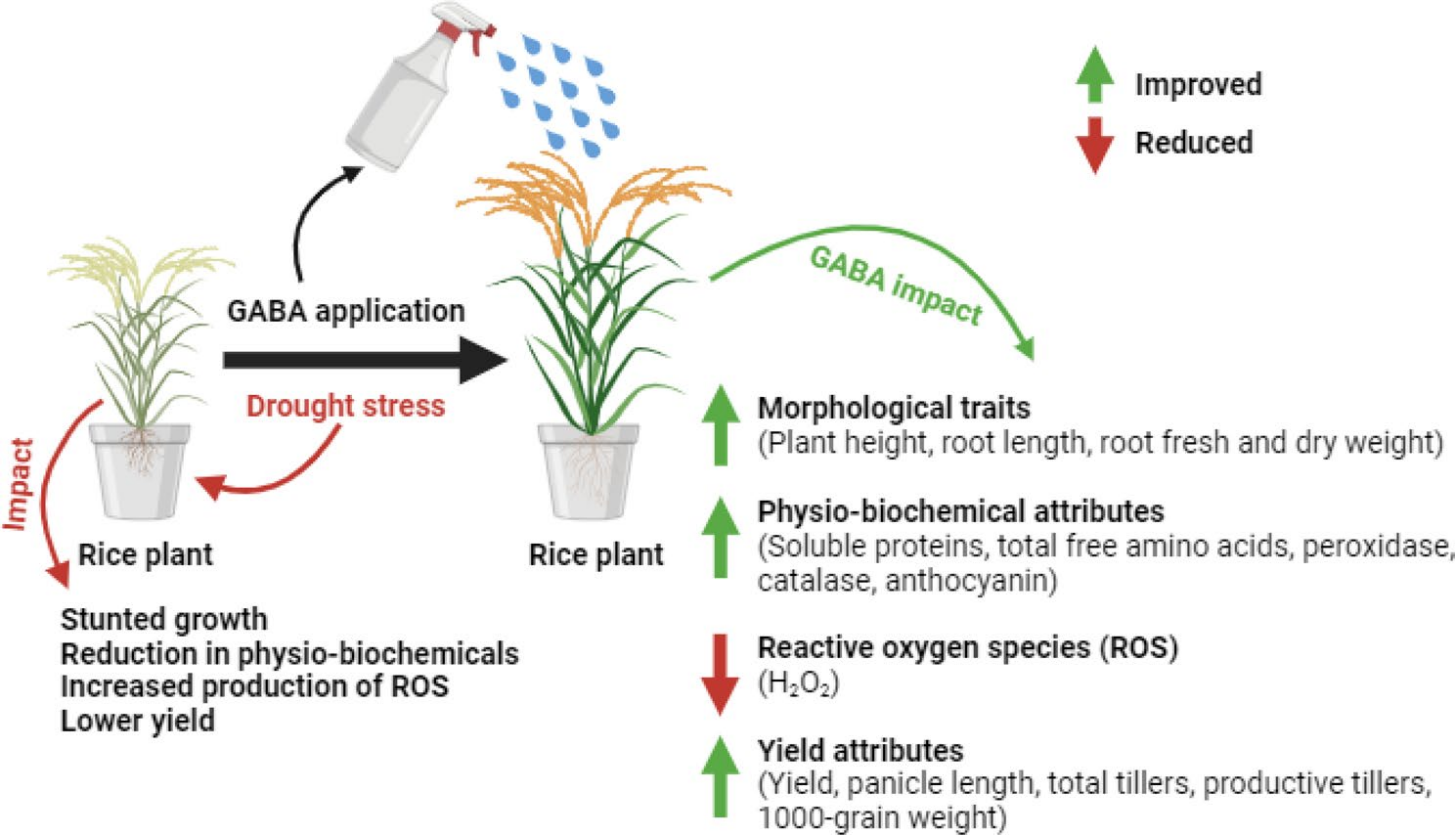
Effect of foliar application of GABA on the morpho-biological and yield attributes of fragrant rice plants under well-watered (FC100) and water stress (FC50) conditions

The overall effect of foliar application of GABA is summarized in Fig. 7, which shows that GABA significantly improved morphological, physio-biochemical, and yield traits in rice. However, further research is essential to gain a deeper understanding about the exact role of GABA in rice physiology and its underlying mechanisms for regulating responses under drought stress. This underscores the need for continued exploration to fully elucidate the pathways through which GABA enhances drought tolerance in fragrant rice.

## Conclusion

Foliar application of GABA significantly improved the performance of fragrant rice plants under well-watered and drought conditions. GABA effectively reduced oxidative stress by modulating antioxidant enzyme activities, leading to improved plant resilience. Additionally, GABA application enhanced yield and yield attributes in both fragrant rice cultivars, with Basmati 515 exhibiting superior performance compared to Super Basmati under drought conditions. Overall, GABA application improved growth, physiological health, and yield of fragrant rice plants in both optimal and water-limited conditions. However, further research is needed to elucidate the precise cellular and molecular mechanisms through which GABA exerts its beneficial effects on fragrant rice plants under drought conditions.

### Electronic supplementary material

Below is the link to the electronic supplementary material.

Supplementary Material 1

## Data Availability

All data generated or analyzed during this study are included in this article.
