# Supplementary material for: Gamma amino butyric acid (GABA) application modulated the morpho-physiological and yield traits of fragrant rice under well-watered and drought conditions
Source: BMC Plant Biol. 2024 Jun 18;24:569. doi: 10.1186/s12870-024-05272-5 (PMC11184787; doi:10.1186/s12870-024-05272-5)
Supplement: Supplementary file 1 — Supplementary Material 1 [file 12870_2024_5272_MOESM1_ESM.docx]

| **Rice varieties** | **Water levels** | **GABA** | **R1** | **R2** | **R3** | **Means** |
| --- | --- | --- | --- | --- | --- | --- |
| **Super Basmati** | **Well-watered** | **Ck** | 83.00 | 83.19 | 83.16 | 83.12 |
|  |  | **G1** | 84.00 | 84.23 | 84.12 | 84.12 |
|  |  | **G2** | 85.00 | 84.25 | 84.90 | 84.72 |
|  |  | **G3** | 85.18 | 85.13 | 85.10 | 85.14 |
|  | **Drought** | **Ck** | 81.67 | 81.23 | 81.67 | 81.52 |
|  |  | **G1** | 82.20 | 81.82 | 82.26 | 82.09 |
|  |  | **G2** | 83.12 | 82.58 | 82.88 | 82.86 |
|  |  | **G3** | 84.13 | 83.51 | 83.66 | 83.77 |
| **Basmati-515** | **Well-watered** | **Ck** | 84.94 | 84.98 | 85.01 | 84.98 |
|  |  | **G1** | 85.14 | 85.16 | 85.47 | 85.26 |
|  |  | **G2** | 85.23 | 85.64 | 85.43 | 85.43 |
|  |  | **G3** | 86.68 | 86.82 | 86.55 | 86.68 |
|  | **Drought** | **Ck** | 82.13 | 82.26 | 82.27 | 82.22 |
|  |  | **G1** | 83.15 | 83.26 | 83.29 | 83.23 |
|  |  | **G2** | 84.12 | 84.35 | 84.20 | 84.22 |
|  |  | **G3** | 85.14 | 85.18 | 84.90 | 85.07 |

**Table S1: Effects of foliar application of GABA on plant height of two fragrant rice cultivars (Super Basmati and Basmati-515) under well-watered (FC100) and drought (FC50) conditions**

**Table S2: Effects of foliar application of GABA on root length of two fragrant rice cultivars (Super Basmati and Basmati-515) under well-watered (FC100) and drought (FC50) conditions**

| **Rice varieties** | **Water levels** | **GABA** | **R1** | **R2** | **R3** | **Means** |
| --- | --- | --- | --- | --- | --- | --- |
| **Super Basmati** | **Well-watered** | **Ck** | 8.68 | 7.30 | 8.10 | 8.03 |
|  |  | **G1** | 11.00 | 13.86 | 11.00 | 11.95 |
|  |  | **G2** | 12.06 | 13.50 | 11.34 | 12.30 |
|  |  | **G3** | 12.96 | 13.00 | 13.02 | 12.99 |
|  | **Drought** | **Ck** | 5.00 | 6.00 | 6.98 | 5.99 |
|  |  | **G1** | 6.41 | 7.39 | 7.30 | 7.03 |
|  |  | **G2** | 10.00 | 11.00 | 9.21 | 10.07 |
|  |  | **G3** | 10.78 | 11.08 | 11.00 | 10.95 |
| **Basmati-515** | **Well-watered** | **Ck** | 10.64 | 11.00 | 9.94 | 10.53 |
|  |  | **G1** | 12.87 | 11.80 | 12.00 | 12.22 |
|  |  | **G2** | 13.40 | 13.10 | 12.99 | 13.16 |
|  |  | **G3** | 14.23 | 13.90 | 14.63 | 14.25 |
|  | **Drought** | **Ck** | 7.00 | 7.24 | 6.97 | 7.07 |
|  |  | **G1** | 9.23 | 9.00 | 9.89 | 9.37 |
|  |  | **G2** | 11.20 | 10.29 | 12.00 | 11.16 |
|  |  | **G3** | 12.00 | 11.45 | 13.10 | 12.18 |

**Table S3: Effects of foliar application of GABA on root fresh weight of two fragrant rice cultivars (Super Basmati and Basmati-515) under well-watered (FC100) and drought (FC50) conditions**

| **Rice varieties** | **Water levels** | **GABA** | **R1** | **R2** | **R3** | **Means** |
| --- | --- | --- | --- | --- | --- | --- |
| Super Basmati | **Well-watered** | **Ck** | 20.00 | 19.51 | 20.49 | 20.00 |
|  |  | **G1** | 23.72 | 23.98 | 23.59 | 23.76 |
|  |  | **G2** | 25.50 | 25.37 | 26.01 | 25.63 |
|  |  | **G3** | 27.00 | 27.48 | 26.54 | 27.01 |
|  | **Drought** | **Ck** | 16.00 | 16.07 | 15.95 | 16.01 |
|  |  | **G1** | 19.66 | 19.33 | 20.01 | 19.67 |
|  |  | **G2** | 21.72 | 21.59 | 21.85 | 21.72 |
|  |  | **G3** | 23.00 | 22.91 | 23.09 | 23.00 |
| **Basmati-515** | **Well-watered** | **Ck** | 22.00 | 21.95 | 22.05 | 22.00 |
|  |  | **G1** | 29.55 | 30.01 | 29.16 | 29.57 |
|  |  | **G2** | 34.00 | 33.89 | 34.11 | 34.00 |
|  |  | **G3** | 29.55 | 30.01 | 29.16 | 29.57 |
|  | **Drought** | **Ck** | 18.00 | 17.86 | 18.15 | 18.00 |
|  |  | **G1** | 21.31 | 22.06 | 21.05 | 21.47 |
|  |  | **G2** | 23.00 | 23.09 | 22.91 | 23.00 |
|  |  | **G3** | 25.77 | 26.23 | 25.46 | 25.82 |

**Table S4: Effects of foliar application of GABA on root dry weight of two fragrant rice cultivars (Super Basmati and Basmati-515) under well-watered (FC100) and drought (FC50) conditions**

| **Rice varieties** | **Water levels** | **GABA** | **R1** | **R2** | **R3** | **Means** |
| --- | --- | --- | --- | --- | --- | --- |
| **Super Basmati** | **Well-watered** | **Ck** | 8.02 | 8.47 | 8.00 | 8.16 |
|  |  | **G1** | 10.19 | 10.29 | 10.13 | 10.20 |
|  |  | **G2** | 11.56 | 12.03 | 11.11 | 11.57 |
|  |  | **G3** | 12.67 | 12.95 | 12.39 | 12.67 |
|  | **Drought** | **Ck** | 6.64 | 6.71 | 6.58 | 6.64 |
|  |  | **G1** | 8.87 | 9.03 | 8.73 | 8.88 |
|  |  | **G2** | 10.00 | 10.39 | 9.81 | 10.07 |
|  |  | **G3** | 11.20 | 11.55 | 10.87 | 11.21 |
| **Basmati-515** | **Well-watered** | **Ck** | 12.00 | 12.27 | 11.75 | 12.01 |
|  |  | **G1** | 13.67 | 13.37 | 13.98 | 13.67 |
|  |  | **G2** | 15.00 | 14.51 | 15.51 | 15.01 |
|  |  | **G3** | 16.54 | 16.11 | 16.98 | 16.54 |
|  | **Drought** | **Ck** | 10.00 | 9.58 | 10.43 | 10.00 |
|  |  | **G1** | 10.31 | 9.93 | 10.69 | 10.31 |
|  |  | **G2** | 12.65 | 12.45 | 12.86 | 12.65 |
|  |  | **G3** | 13.64 | 13.86 | 13.42 | 13.64 |

**Table S5: Effects of foliar application of GABA on total soluble proteins of two fragrant rice cultivars (Super Basmati and Basmati-515) under well-watered (FC100) and drought (FC50) conditions**

| **Rice varieties** | **Water levels** | **GABA** | **R1** | **R2** | **R3** | **Means** |
| --- | --- | --- | --- | --- | --- | --- |
| **Super Basmati** | **Well-watered** | **Ck** | 4.32 | 4.21 | 4.45 | 4.33 |
|  |  | **G1** | 5.00 | 5.09 | 4.99 | 5.02 |
|  |  | **G2** | 5.31 | 5.12 | 5.19 | 5.20 |
|  |  | **G3** | 5.32 | 5.53 | 5.51 | 5.45 |
|  | **Drought** | **Ck** | 3.05 | 3.09 | 3.02 | 3.05 |
|  |  | **G1** | 4.32 | 4.21 | 4.41 | 4.31 |
|  |  | **G2** | 4.75 | 4.68 | 4.50 | 4.64 |
|  |  | **G3** | 4.87 | 4.77 | 4.92 | 4.85 |
| **Basmati-515** | **Well-watered** | **Ck** | 5.00 | 5.23 | 5.21 | 5.15 |
|  |  | **G1** | 5.31 | 5.52 | 5.27 | 5.37 |
|  |  | **G2** | 5.88 | 5.82 | 5.72 | 5.81 |
|  |  | **G3** | 5.91 | 6.08 | 5.94 | 5.98 |
|  | **Drought** | **Ck** | 3.89 | 3.78 | 3.99 | 3.89 |
|  |  | **G1** | 4.54 | 4.68 | 4.81 | 4.68 |
|  |  | **G2** | 4.86 | 5.00 | 5.21 | 5.02 |
|  |  | **G3** | 4.94 | 5.10 | 5.12 | 5.05 |

**Table S6: Effects of foliar application of GABA on total free amino acids of two fragrant rice cultivars (Super Basmati and Basmati-515) under well-watered (FC100) and drought (FC50) conditions**

| **Rice varieties** | **Water levels** | **GABA** | **R1** | **R2** | **R3** | **Means** |
| --- | --- | --- | --- | --- | --- | --- |
| **Super Basmati** | **Well-watered** | **Ck** | 2.43 | 2.51 | 2.55 | 2.50 |
|  |  | **G1** | 2.99 | 3.06 | 3.11 | 3.05 |
|  |  | **G2** | 3.59 | 3.63 | 3.66 | 3.63 |
|  |  | **G3** | 3.99 | 4.06 | 4.12 | 4.05 |
|  | **Drought** | **Ck** | 1.52 | 1.57 | 1.58 | 1.56 |
|  |  | **G1** | 1.64 | 1.64 | 1.70 | 1.66 |
|  |  | **G2** | 1.73 | 1.73 | 1.85 | 1.77 |
|  |  | **G3** | 2.49 | 2.56 | 2.60 | 2.55 |
| **Basmati-515** | **Well-watered** | **Ck** | 2.82 | 2.88 | 2.94 | 2.88 |
|  |  | **G1** | 3.49 | 3.55 | 3.61 | 3.55 |
|  |  | **G2** | 4.01 | 4.07 | 4.13 | 4.07 |
|  |  | **G3** | 4.38 | 4.43 | 4.49 | 4.43 |
|  | **Drought** | **Ck** | 2.04 | 2.08 | 2.11 | 2.08 |
|  |  | **G1** | 1.92 | 1.99 | 2.04 | 1.99 |
|  |  | **G2** | 2.42 | 2.43 | 2.50 | 2.45 |
|  |  | **G3** | 2.66 | 2.43 | 2.79 | 2.63 |

**Table S7: Effects of foliar application of GABA on hydrogen peroxide of two fragrant rice cultivars (Super Basmati and Basmati-515) under well-watered (FC100) and drought (FC50) conditions**

| **Rice varieties** | **Water levels** | **GABA** | **R1** | **R2** | **R3** | **Means** |
| --- | --- | --- | --- | --- | --- | --- |
| **Super Basmati** | **Well-watered** | **Ck** | 1.15 | 1.26 | 1.13 | 1.18 |
|  |  | **G1** | 2.30 | 2.10 | 1.99 | 2.13 |
|  |  | **G2** | 2.24 | 2.33 | 2.26 | 2.28 |
|  |  | **G3** | 2.39 | 2.19 | 2.16 | 2.25 |
|  | **Drought** | **Ck** | 1.50 | 1.60 | 1.41 | 1.50 |
|  |  | **G1** | 2.70 | 2.50 | 2.43 | 2.54 |
|  |  | **G2** | 3.00 | 2.80 | 2.79 | 2.86 |
|  |  | **G3** | 3.30 | 3.15 | 3.25 | 3.23 |
| **Basmati-515** | **Well-watered** | **Ck** | 0.74 | 0.94 | 0.87 | 0.85 |
|  |  | **G1** | 1.76 | 1.54 | 1.61 | 1.64 |
|  |  | **G2** | 1.89 | 2.00 | 1.97 | 1.95 |
|  |  | **G3** | 2.00 | 2.25 | 2.21 | 2.15 |
|  | **Drought** | **Ck** | 2.00 | 2.30 | 2.29 | 2.20 |
|  |  | **G1** | 3.00 | 3.18 | 3.15 | 3.11 |
|  |  | **G2** | 3.10 | 3.25 | 3.17 | 3.17 |
|  |  | **G3** | 3.59 | 3.48 | 3.79 | 3.62 |

**Table S8: Effects of foliar application of GABA on peroxidase of two fragrant rice cultivars (Super Basmati and Basmati-515) under well-watered (FC100) and drought (FC50) conditions**

| **Rice varieties** | **Water levels** | **GABA** | **R1** | **R2** | **R3** | **Means** |
| --- | --- | --- | --- | --- | --- | --- |
| **Super Basmati** | **Well-watered** | **Ck** | 15.00 | 15.20 | 15.60 | 15.27 |
|  |  | **G1** | 16.00 | 16.30 | 16.15 | 16.15 |
|  |  | **G2** | 17.74 | 17.80 | 18.00 | 17.85 |
|  |  | **G3** | 19.00 | 19.56 | 19.70 | 19.42 |
|  | **Drought** | **Ck** | 13.74 | 14.00 | 14.00 | 13.91 |
|  |  | **G1** | 14.20 | 15.00 | 15.30 | 14.83 |
|  |  | **G2** | 15.40 | 16.10 | 16.20 | 15.90 |
|  |  | **G3** | 16.00 | 17.00 | 17.34 | 16.78 |
| **Basmati-515** | **Well-watered** | **Ck** | 17.00 | 17.20 | 17.45 | 17.22 |
|  |  | **G1** | 18.00 | 18.57 | 18.46 | 18.34 |
|  |  | **G2** | 19.10 | 19.30 | 19.35 | 19.25 |
|  |  | **G3** | 20.00 | 21.00 | 20.34 | 20.45 |
|  | **Drought** | **Ck** | 14.26 | 15.00 | 14.70 | 14.65 |
|  |  | **G1** | 15.00 | 16.00 | 15.90 | 15.63 |
|  |  | **G2** | 15.80 | 16.30 | 16.48 | 16.19 |
|  |  | **G3** | 16.90 | 16.70 | 17.00 | 16.87 |

**Table S9: Effects of foliar application of GABA on catalase of two fragrant rice cultivars (Super Basmati and Basmati-515) under well-watered (FC100) and drought (FC50) conditions**

| **Rice varieties** | **Water levels** | **GABA** | **R1** | **R2** | **R3** | **Means** |
| --- | --- | --- | --- | --- | --- | --- |
| **Super Basmati** | **Well-watered** | **Ck** | 0.45 | 0.47 | 0.48 | 0.47 |
|  |  | **G1** | 0.77 | 0.79 | 0.79 | 0.78 |
|  |  | **G2** | 0.95 | 0.96 | 0.96 | 0.95 |
|  |  | **G3** | 1.43 | 1.51 | 1.60 | 1.51 |
|  | **Drought** | **Ck** | 0.12 | 0.13 | 0.14 | 0.13 |
|  |  | **G1** | 0.35 | 0.36 | 0.37 | 0.36 |
|  |  | **G2** | 0.44 | 0.45 | 0.46 | 0.45 |
|  |  | **G3** | 0.56 | 0.57 | 0.56 | 0.56 |
| **Basmati-515** | **Well-watered** | **Ck** | 0.77 | 0.76 | 0.76 | 0.76 |
|  |  | **G1** | 1.45 | 1.46 | 1.46 | 1.46 |
|  |  | **G2** | 1.69 | 1.70 | 1.71 | 1.70 |
|  |  | **G3** | 1.89 | 1.91 | 1.91 | 1.90 |
|  | **Drought** | **Ck** | 0.30 | 0.31 | 0.32 | 0.31 |
|  |  | **G1** | 0.59 | 0.60 | 0.61 | 0.60 |
|  |  | **G2** | 0.71 | 0.71 | 0.70 | 0.71 |
|  |  | **G3** | 0.94 | 0.94 | 0.95 | 0.95 |

**Table S10: Effects of foliar application of GABA on anthocyanin of two fragrant rice cultivars (Super Basmati and Basmati-515) under well-watered (FC100) and drought (FC50) conditions**

| **Rice varieties** | **Water levels** | **GABA** | **R1** | **R2** | **R3** | **Means** |
| --- | --- | --- | --- | --- | --- | --- |
| **Super Basmati** | **Well-watered** | **Ck** | 5.22 | 5.37 | 5.48 | 5.36 |
|  |  | **G1** | 6.44 | 8.12 | 8.35 | 7.64 |
|  |  | **G2** | 7.84 | 9.19 | 9.23 | 8.75 |
|  |  | **G3** | 9.22 | 9.27 | 9.31 | 9.27 |
|  | **Drought** | **Ck** | 1.89 | 1.91 | 2.39 | 2.06 |
|  |  | **G1** | 2.25 | 2.35 | 3.27 | 2.62 |
|  |  | **G2** | 4.40 | 4.79 | 5.34 | 4.84 |
|  |  | **G3** | 5.08 | 5.60 | 5.90 | 5.52 |
| **Basmati-515** | **Well-watered** | **Ck** | 7.44 | 8.13 | 8.33 | 7.97 |
|  |  | **G1** | 9.32 | 9.40 | 9.70 | 9.47 |
|  |  | **G2** | 9.64 | 9.57 | 9.81 | 9.68 |
|  |  | **G3** | 9.94 | 10.10 | 10.02 | 10.02 |
|  | **Drought** | **Ck** | 5.16 | 3.67 | 3.95 | 4.26 |
|  |  | **G1** | 5.37 | 5.37 | 5.35 | 5.36 |
|  |  | **G2** | 5.60 | 5.44 | 5.39 | 5.48 |
|  |  | **G3** | 5.75 | 5.57 | 5.51 | 5.61 |

**Table S11: Effects of foliar application of GABA on total tillers plant^-1^ of two fragrant rice cultivars (Super Basmati and Basmati-515) under well-watered (FC100) and drought (FC50) conditions**

| **Rice varieties** | **Water levels** | **GABA** | **R1** | **R2** | **R3** | **Means** |
| --- | --- | --- | --- | --- | --- | --- |
| **Super Basmati** | **Well-watered** | **Ck** | 21.67 | 23.00 | 24.67 | 23.11 |
|  |  | **G1** | 27.00 | 28.67 | 30.67 | 28.78 |
|  |  | **G2** | 30.67 | 30.00 | 33.67 | 31.44 |
|  |  | **G3** | 33.67 | 33.67 | 35.17 | 34.17 |
|  | **Drought** | **Ck** | 20.67 | 19.33 | 19.76 | 19.92 |
|  |  | **G1** | 23.67 | 24.98 | 23.70 | 24.12 |
|  |  | **G2** | 24.00 | 25.00 | 28.17 | 25.72 |
|  |  | **G3** | 29.67 | 25.33 | 28.89 | 27.96 |
| **Basmati-515** | **Well-watered** | **Ck** | 29.67 | 26.78 | 28.78 | 28.41 |
|  |  | **G1** | 33.33 | 33.45 | 31.56 | 32.78 |
|  |  | **G2** | 34.67 | 34.33 | 33.00 | 34.00 |
|  |  | **G3** | 38.00 | 40.73 | 38.87 | 39.20 |
|  | **Drought** | **Ck** | 20.00 | 19.00 | 20.07 | 19.69 |
|  |  | **G1** | 22.33 | 25.30 | 27.34 | 24.99 |
|  |  | **G2** | 22.33 | 27.27 | 23.33 | 24.31 |
|  |  | **G3** | 23.00 | 26.67 | 26.56 | 25.41 |

**Table S12: Effects of foliar application of GABA on productive tillers plant^-1^ of two fragrant rice cultivars (Super Basmati and Basmati-515) under well-watered (FC100) and drought (FC50) conditions**

| **Rice varieties** | **Water levels** | **GABA** | **R1** | **R2** | **R3** | **Means** |
| --- | --- | --- | --- | --- | --- | --- |
| **Super Basmati** | **Well-watered** | **Ck** | 12.66 | 15.00 | 16.66 | 14.77 |
|  |  | **G1** | 19.00 | 20.66 | 21.66 | 20.44 |
|  |  | **G2** | 21.66 | 22.00 | 23.66 | 22.44 |
|  |  | **G3** | 23.66 | 24.66 | 24.66 | 24.33 |
|  | **Drought** | **Ck** | 5.66 | 5.33 | 4.00 | 5.00 |
|  |  | **G1** | 9.66 | 10.33 | 8.00 | 9.33 |
|  |  | **G2** | 10.00 | 9.00 | 11.66 | 10.22 |
|  |  | **G3** | 14.66 | 8.33 | 11.33 | 11.44 |
| **Basmati-515** | **Well-watered** | **Ck** | 20.66 | 20.00 | 20.00 | 20.22 |
|  |  | **G1** | 25.33 | 25.00 | 24.00 | 24.78 |
|  |  | **G2** | 27.66 | 26.33 | 27.00 | 27.00 |
|  |  | **G3** | 32.00 | 33.33 | 33.66 | 33.00 |
|  | **Drought** | **Ck** | 8.00 | 6.00 | 7.66 | 7.22 |
|  |  | **G1** | 11.33 | 15.00 | 16.66 | 14.33 |
|  |  | **G2** | 12.33 | 17.33 | 15.33 | 15.00 |
|  |  | **G3** | 15.00 | 17.66 | 18.66 | 17.11 |

**Table S13: Effects of foliar application of GABA on non-productive tillers plant^-1^ of two fragrant rice cultivars (Super Basmati and Basmati-515) under well-watered (FC100) and drought (FC50) conditions**

| **Rice varieties** | **Water levels** | **GABA** | **R1** | **R2** | **R3** | **Means** |
| --- | --- | --- | --- | --- | --- | --- |
| **Super Basmati** | **Well-watered** | **Ck** | 9.00 | 8.00 | 8.00 | 8.33 |
|  |  | **G1** | 8.00 | 8.00 | 9.00 | 8.33 |
|  |  | **G2** | 9.00 | 8.00 | 10.00 | 9.00 |
|  |  | **G3** | 10.00 | 9.00 | 10.50 | 9.83 |
|  | **Drought** | **Ck** | 15.00 | 14.00 | 15.76 | 14.92 |
|  |  | **G1** | 14.00 | 14.65 | 15.70 | 14.78 |
|  |  | **G2** | 14.00 | 16.00 | 16.50 | 15.50 |
|  |  | **G3** | 15.00 | 17.00 | 17.56 | 16.52 |
| **Basmati-515** | **Well-watered** | **Ck** | 9.00 | 9.45 | 8.78 | 9.08 |
|  |  | **G1** | 8.00 | 8.45 | 7.56 | 8.00 |
|  |  | **G2** | 7.00 | 8.00 | 6.00 | 7.00 |
|  |  | **G3** | 6.00 | 7.40 | 5.20 | 6.20 |
|  | **Drought** | **Ck** | 12.00 | 13.00 | 12.40 | 12.47 |
|  |  | **G1** | 11.00 | 10.30 | 10.67 | 10.66 |
|  |  | **G2** | 10.00 | 9.60 | 8.00 | 9.20 |
|  |  | **G3** | 8.00 | 9.00 | 7.89 | 8.30 |

**Table S14: Effects of foliar application of GABA on panicle length of two fragrant rice cultivars (Super Basmati and Basmati-515) under well-watered (FC100) and drought (FC50) conditions**

| **Rice varieties** | **Water levels** | **GABA** | **R1** | **R2** | **R3** | **Means** |
| --- | --- | --- | --- | --- | --- | --- |
| **Super Basmati** | **Well-watered** | **Ck** | 22.33 | 23.00 | 22.33 | 22.56 |
|  |  | **G1** | 23.00 | 22.00 | 24.00 | 23.00 |
|  |  | **G2** | 24.00 | 22.00 | 25.33 | 23.78 |
|  |  | **G3** | 25.00 | 26.50 | 24.00 | 25.17 |
|  | **Drought** | **Ck** | 20.00 | 19.00 | 18.00 | 19.00 |
|  |  | **G1** | 21.00 | 20.67 | 21.00 | 20.89 |
|  |  | **G2** | 21.50 | 19.00 | 20.00 | 20.17 |
|  |  | **G3** | 23.00 | 22.33 | 24.00 | 23.11 |
| **Basmati-515** | **Well-watered** | **Ck** | 23.00 | 24.00 | 25.00 | 24.00 |
|  |  | **G1** | 24.00 | 25.55 | 26.00 | 25.18 |
|  |  | **G2** | 25.56 | 26.00 | 27.00 | 26.19 |
|  |  | **G3** | 27.00 | 28.00 | 29.00 | 28.00 |
|  | **Drought** | **Ck** | 20.22 | 21.00 | 22.00 | 21.07 |
|  |  | **G1** | 21.00 | 22.20 | 21.00 | 21.40 |
|  |  | **G2** | 20.33 | 22.56 | 21.00 | 21.30 |
|  |  | **G3** | 22.00 | 23.00 | 24.00 | 23.00 |

**Table S15: Effects of foliar application of GABA on branches panicle^-1^ of two fragrant rice cultivars (Super Basmati and Basmati-515) under well-watered (FC100) and drought (FC50) conditions**

| **Rice varieties** | **Water levels** | **GABA** | **R1** | **R2** | **R3** | **Means** |
| --- | --- | --- | --- | --- | --- | --- |
| **Super Basmati** | **Well-watered** | **Ck** | 7.89 | 7.76 | 7.76 | 7.80 |
|  |  | **G1** | 9.79 | 9.02 | 9.90 | 9.57 |
|  |  | **G2** | 10.46 | 10.46 | 10.61 | 10.51 |
|  |  | **G3** | 11.02 | 11.11 | 11.21 | 11.11 |
|  | **Drought** | **Ck** | 4.35 | 4.42 | 4.51 | 4.43 |
|  |  | **G1** | 5.47 | 5.59 | 5.65 | 5.57 |
|  |  | **G2** | 7.68 | 7.76 | 7.62 | 7.69 |
|  |  | **G3** | 8.97 | 8.93 | 8.99 | 8.96 |
| **Basmati-515** | **Well-watered** | **Ck** | 9.70 | 9.40 | 9.18 | 9.43 |
|  |  | **G1** | 11.35 | 11.47 | 11.52 | 11.45 |
|  |  | **G2** | 12.33 | 12.00 | 11.33 | 11.89 |
|  |  | **G3** | 15.00 | 15.04 | 15.11 | 15.05 |
|  | **Drought** | **Ck** | 7.77 | 7.85 | 7.25 | 7.62 |
|  |  | **G1** | 8.15 | 8.26 | 8.35 | 8.25 |
|  |  | **G2** | 9.00 | 9.07 | 9.19 | 9.09 |
|  |  | **G3** | 10.00 | 10.22 | 10.36 | 10.19 |

**Table S16: Effects of foliar application of GABA on grains panicle^-1^ of two fragrant rice cultivars (Super Basmati and Basmati-515) under well-watered (FC100) and drought (FC50) conditions**

| **Rice varieties** | **Water levels** | **GABA** | **R1** | **R2** | **R3** | **Means** |
| --- | --- | --- | --- | --- | --- | --- |
| **Super Basmati** | **Well-watered** | **Ck** | 52.00 | 53.00 | 54.00 | 53.00 |
|  |  | **G1** | 54.00 | 53.00 | 51.00 | 52.67 |
|  |  | **G2** | 55.00 | 55.65 | 54.34 | 55.00 |
|  |  | **G3** | 56.00 | 57.00 | 57.75 | 56.92 |
|  | **Drought** | **Ck** | 41.00 | 40.00 | 39.00 | 40.00 |
|  |  | **G1** | 43.00 | 45.00 | 46.00 | 44.67 |
|  |  | **G2** | 46.00 | 48.00 | 50.00 | 48.00 |
|  |  | **G3** | 52.00 | 53.00 | 49.00 | 51.33 |
| **Basmati-515** | **Well-watered** | **Ck** | 56.00 | 58.00 | 60.00 | 58.00 |
|  |  | **G1** | 59.00 | 60.00 | 62.00 | 60.33 |
|  |  | **G2** | 61.00 | 64.00 | 66.00 | 63.67 |
|  |  | **G3** | 66.00 | 68.00 | 69.00 | 67.67 |
|  | **Drought** | **Ck** | 45.66 | 43.00 | 41.40 | 43.35 |
|  |  | **G1** | 44.00 | 45.20 | 47.45 | 45.55 |
|  |  | **G2** | 47.00 | 50.00 | 53.00 | 50.00 |
|  |  | **G3** | 54.00 | 55.00 | 56.00 | 55.00 |

**Table S17: Effects of foliar application of GABA on 1000-grain weight of two fragrant rice cultivars (Super Basmati and Basmati-515) under well-watered (FC100) and drought (FC50) conditions**

| **Rice varieties** | **Water levels** | **GABA** | **R1** | **R2** | **R3** | **Means** |
| --- | --- | --- | --- | --- | --- | --- |
| **Super Basmati** | **Well-watered** | **Ck** | 10.00 | 9.50 | 10.50 | 10.00 |
|  |  | **G1** | 11.12 | 12.00 | 10.12 | 11.08 |
|  |  | **G2** | 12.87 | 12.37 | 12.87 | 12.70 |
|  |  | **G3** | 13.30 | 11.30 | 14.30 | 12.97 |
|  | **Drought** | **Ck** | 5.00 | 5.50 | 4.50 | 5.00 |
|  |  | **G1** | 5.75 | 5.00 | 6.25 | 5.67 |
|  |  | **G2** | 6.84 | 7.25 | 6.84 | 6.98 |
|  |  | **G3** | 8.00 | 8.50 | 7.00 | 7.83 |
| **Basmati-515** | **Well-watered** | **Ck** | 12.00 | 12.50 | 11.50 | 12.00 |
|  |  | **G1** | 14.52 | 15.00 | 14.00 | 14.51 |
|  |  | **G2** | 15.64 | 16.20 | 15.64 | 15.83 |
|  |  | **G3** | 16.43 | 15.43 | 17.30 | 16.39 |
|  | **Drought** | **Ck** | 8.00 | 7.50 | 8.50 | 8.00 |
|  |  | **G1** | 9.45 | 10.25 | 9.00 | 9.57 |
|  |  | **G2** | 10.34 | 11.00 | 10.00 | 10.45 |
|  |  | **G3** | 11.87 | 12.00 | 11.98 | 11.95 |

**Table S18: Effects of foliar application of GABA on grain yield of two fragrant rice cultivars (Super Basmati and Basmati-515) under well-watered (FC100) and drought (FC50) conditions**

| **Rice varieties** | **Water levels** | **GABA** | **R1** | **R2** | **R3** | **Means** |
| --- | --- | --- | --- | --- | --- | --- |
| **Super Basmati** | **Well-watered** | **Ck** | 10.30 | 10.49 | 10.20 | 10.33 |
|  |  | **G1** | 12.20 | 12.31 | 12.61 | 12.37 |
|  |  | **G2** | 12.86 | 13.00 | 12.90 | 12.92 |
|  |  | **G3** | 13.25 | 13.65 | 13.70 | 13.53 |
|  | **Drought** | **Ck** | 8.21 | 8.52 | 8.10 | 8.28 |
|  |  | **G1** | 9.80 | 9.90 | 10.00 | 9.90 |
|  |  | **G2** | 10.70 | 10.10 | 10.50 | 10.43 |
|  |  | **G3** | 11.00 | 11.78 | 11.20 | 11.33 |
| **Basmati-515** | **Well-watered** | **Ck** | 11.71 | 11.86 | 11.63 | 11.73 |
|  |  | **G1** | 12.43 | 12.54 | 12.84 | 12.60 |
|  |  | **G2** | 13.83 | 13.90 | 13.89 | 13.87 |
|  |  | **G3** | 14.92 | 14.90 | 15.00 | 14.94 |
|  | **Drought** | **Ck** | 9.91 | 9.71 | 9.82 | 9.81 |
|  |  | **G1** | 10.52 | 10.73 | 10.65 | 10.63 |
|  |  | **G2** | 11.51 | 11.61 | 11.42 | 11.51 |
|  |  | **G3** | 12.39 | 12.52 | 12.69 | 12.53 |

**Table S19: Effects of foliar application of GABA on straw yield of two fragrant rice cultivars (Super Basmati and Basmati-515) under well-watered (FC100) and drought (FC50) conditions**

| **Rice varieties** | **Water levels** | **GABA** | **R1** | **R2** | **R3** | **Means** |
| --- | --- | --- | --- | --- | --- | --- |
| **Super Basmati** | **Well-watered** | **Ck** | 90.00 | 91.00 | 89.00 | 90.00 |
|  |  | **G1** | 92.00 | 95.00 | 94.33 | 93.78 |
|  |  | **G2** | 96.66 | 98.00 | 95.00 | 96.55 |
|  |  | **G3** | 101.33 | 103.00 | 100.33 | 101.55 |
|  | **Drought** | **Ck** | 81.00 | 82.00 | 80.00 | 81.00 |
|  |  | **G1** | 83.00 | 84.00 | 81.00 | 82.67 |
|  |  | **G2** | 85.00 | 84.00 | 87.00 | 85.33 |
|  |  | **G3** | 88.00 | 90.00 | 89.00 | 89.00 |
| **Basmati-515** | **Well-watered** | **Ck** | 91.00 | 90.00 | 92.00 | 91.00 |
|  |  | **G1** | 100.33 | 101.66 | 105.55 | 102.51 |
|  |  | **G2** | 102.00 | 106.33 | 102.00 | 103.44 |
|  |  | **G3** | 107.66 | 107.66 | 110.30 | 108.54 |
|  | **Drought** | **Ck** | 82.00 | 83.00 | 84.00 | 83.00 |
|  |  | **G1** | 85.00 | 86.00 | 84.00 | 85.00 |
|  |  | **G2** | 86.00 | 86.00 | 88.00 | 86.67 |
|  |  | **G3** | 91.00 | 92.00 | 94.00 | 92.33 |
